# A fluorescent bead-based multiplex assay for the detection of *Brucella* sp. specific antibodies in canine serum

**DOI:** 10.3389/fmicb.2025.1655877

**Published:** 2025-10-08

**Authors:** Cassandra Guarino, Rebecca Franklin-Guild, Sanda Asbie, Toby Pinn-Woodcock, Anja Serap Sipka, Colleen Eade, Lauren Griggs, Elizabeth Altier, Kristina Ceres, Yrjo Grohn, Craig Altier, Bettina Wagner

**Affiliations:** Department of Population Medicine and Diagnostic Sciences, College of Veterinary Medicine, Cornell University, Ithaca, NY, United States

**Keywords:** canine brucellosis, Luminex, serology, BP26, Omp31, PO1

## Abstract

**Introduction:**

The zoonotic pathogen, *Brucella canis*, causes brucellosis in dogs. Infection with *B. canis* is usually diagnosed by serological testing. We developed a fluorescent bead-based multiplex assay for detection of *B. canis* specific antibodies in canine serum. The assay consists of two antigens detected simultaneously by canine serum antibodies. One antigen, BP26, was selected from a set of immunodominant proteins identified through western blot and proteomics analysis. The second antigen, PO1, is a 17 amino acid peptide derived from *B. canis* Omp31.

**Methods:**

Dog sera from diagnostic submissions were tested in parallel with a reference assay consisting of a rapid slide agglutination test (2ME-RSAT) and an agar gel immunodiffusion test (AGID II). A Bayesian latent class model (BLCM) was utilized to determine sensitivity and specificity of both assays. For the model to be identifiable, two groups with differing prevalence were included; one group was composed of 1,192 diagnostic submissions, and the second group was composed of 390 samples submitted for export purposes.

**Results:**

The seroprevalence of *B. canis* specific antibodies in these two groups was estimated to be 16.1% (95% CI, 12.6–19.3%) and 0.1% (95%, 0.0–0.6%), respectively. Diagnostic sensitivity and specificity of the two-antigen assay for detecting *B. canis* specific antibodies were 91.6% (95% CI, 85.2–98.0%) and 94.9% (95% CI, 92.3–96.9%), respectively.

**Discussion:**

The addition of a third cytoplasmic antigen further increased assay sensitivity. The Canine Brucella Multiplex assay is a novel and quantitative diagnostic tool for detecting *B. canis* antibodies in canine serum to aid in the diagnosis of brucellosis in dogs.

## Introduction

*Brucella canis* is a Gram-negative proteobacterium in the family Brucellaceae that causes brucellosis in canids. This bacterium was first described in 1968 as the causative agent of a series of abortion storms in beagle kennels (Spink and Morisset, 1970; Carmichael and Kenney, 1968) and is known to have zoonotic potential (Marzetti et al., 2013; Polt et al., 1982). Commonly described clinical manifestations of *B. canis* infection in dogs include lymphadenitis, discospondylitis and orchitis/epididymitis (Anderson and Binnington, 1983; Egloff et al., 2018; Kerwin et al., 1992). However, clinical signs of canine brucellosis in dogs are generally nonspecific (Wanke, 2004); often, the only indication of infection in females is spontaneous abortion between gestation days 30 and 57 (Carmichael and Kenney, 1968). *Brucella canis* is suspected to have a prevalence of approximately 5% in the United States dog population and has been identified in many countries around the world (Santos et al., 2021). While prevalence can vary widely with location and population dynamics, frequent outbreaks, transmission, and dissemination could be avoided with appropriate testing and quarantine procedures (Hensel et al., 2018; Hollett, 2006). Outbreaks of *B. canis* have the potential to impact human health and cause substantial economic losses in the dog breeding industry.

The “gold standard” test used to confirm infection with *B. canis* is culture of the bacteria from blood or tissue. However, episodes of bacteremia can be intermittent, leading to low sensitivity of bacterial culture and frequent false-negative results (Greene and Carmichael, 2012). In addition, culturing *B. canis* bacteria presents difficulties as organisms are fastidious and slow-growing (Yagupsky, 1999). Therefore, serologic assays that detect *B. canis* antibodies have been developed to find evidence of *B. canis* infection in dogs. Traditional serologic assays include rapid slide agglutination test (RSAT), tube agglutination test (TAT), Agar gel immunodiffusion assay (AGID), immunofluorescence antibody test (IFA), or several different enzyme linked immunosorbent assays (ELISAs) (Greene and Carmichael, 2012; Keid et al., 2009; Mol et al., 2020; Lucero et al., 2002). The available serologic *B. canis* assays all have the potential for cross-reactivity with shared surface antigens of other Gram-negative bacteria (e.g., *Pseudomonas aeruginosa*, *Bordetella bronchiseptica,* and *Yersinia* spp.) leading to false-positive assay results (Hollett, 2006; Greene and Carmichael, 2012). Therefore, screening tests such as RSAT, TAT, ELISA, or IFA must be followed by a reference assay for confirmation. One method to confirm a positive result from a screening test is to pre-treat serum with 2-mercaptoethanol (2ME) to dissociate IgM, which is more likely than IgG to cross-react with related bacteria (Woods, 2013; Buchanan and Faber, 1980). As with any serologic assay, false-negative assay results can occur in the early stage of infection, prior to seroconversion, and in the case of *B. canis*, some chronically infected dogs become seronegative (Wanke, 2004; Greene and Carmichael, 2012; Wooley et al., 1978).

A commonly used reference assay is the combination of a 2ME rapid slide agglutination test (2ME-RSAT) and an agar gel immunodiffusion test (AGID) assay that employs the cytoplasmic components of *B. canis* (AGID II). Antigens used for both these tests are produced from a strain of *B. canis* that is less mucoid (M-), allowing for increased sensitivity as well as fewer non-specific reactions (Carmichael et al., 1984a). This combination of tests is a preferred reference assay due to the exceptionally high specificity; the specificity of the AGID II assay is estimated to be >99% (Keid et al., 2009; Carmichael et al., 1984b). However, there are several challenges associated with the production of the 2ME-RSAT/AGID II reference assay. Both tests require regular growth of *B. canis*, a zoonotic pathogen handled at biosafety level 3 (BSL3), to produce antigen for the assays. In addition, the AGID II assay requires large amounts of characterized control serum from dogs with known history of *B. canis* infection—ideally from exsanguination of an experimentally infected specific pathogen free (SPF) dog, which can be challenging to obtain. Furthermore, the interpretation of AGID II assay results cannot be automated and, therefore, can be subjective.

Our objective was to create a novel serologic assay to support the diagnosis of *B. canis* infection in dogs that could overcome the challenges associated with current reference assays for *B. canis* diagnosis. Here we describe a set of recombinant antigens that can be employed in a multiplex assay format and manufactured without the risk of human laboratory exposure or need for substantial quantities of positive control canine serum, produces objective and quantitative results, and provides reasonable sensitivity and specificity for the serologic diagnosis of *B. canis* infection.

## Materials and methods

### Serum samples

**Figure 1:**
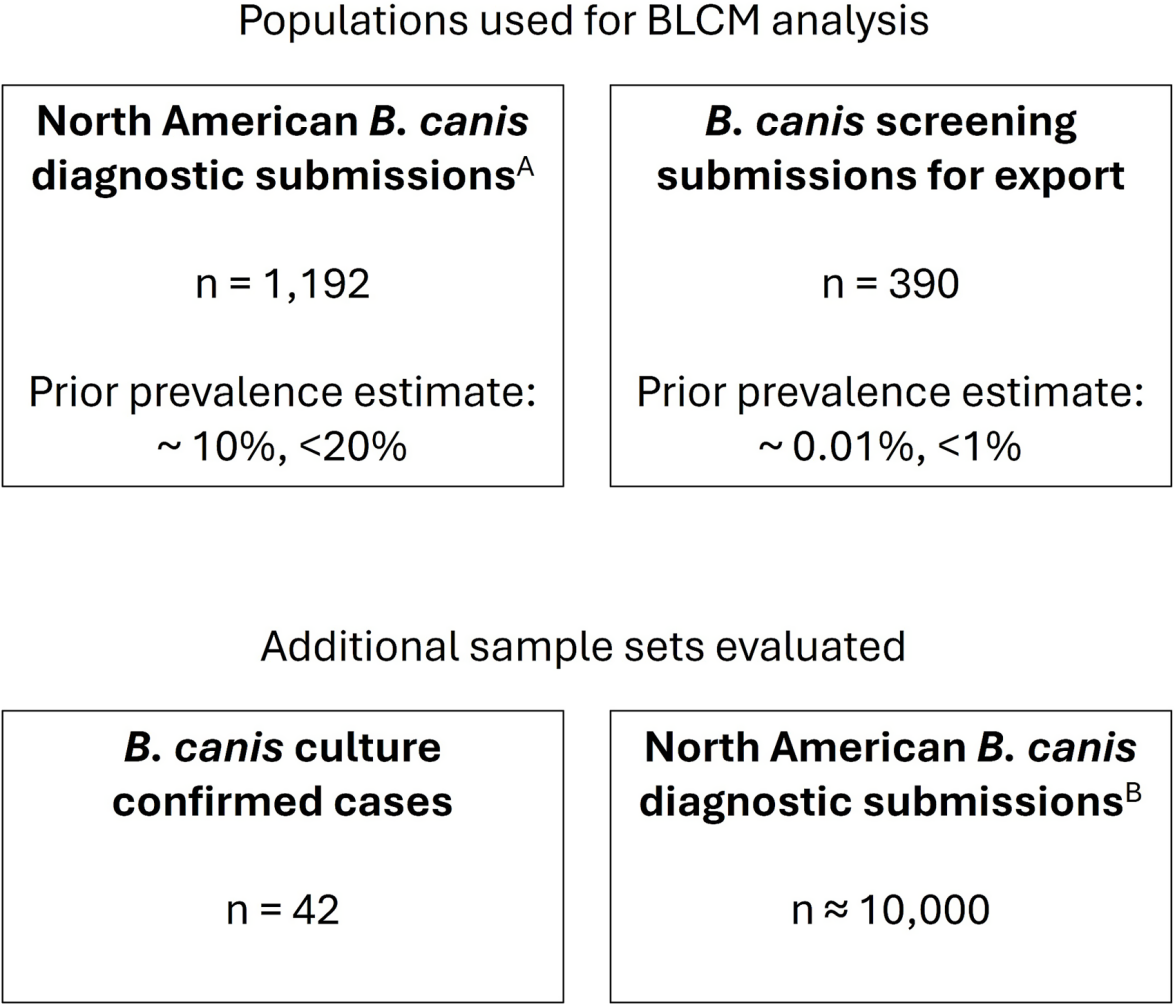
Serum samples evaluated. Serum samples were derived from routine diagnostic submissions to the AHDC. ^A^Subset of samples submitted 08/2020–08/2022. ^B^Samples submitted 01/2023–12/2024.

All canine serum samples were submitted by veterinary practitioners for *B. canis* serological testing to the Animal Health Diagnostic Center (AHDC) at Cornell University (Figure 1). The samples were tested by both the 2ME-RSAT and the AGID II assay. The 2ME-RSAT is performed with heat-killed whole-cell *B. canis* (M-) antigen, and the AGID II is performed with *B. canis* (M-) cytoplasmic antigen, as described below.

A total of 1,582 diagnostic serum sample submissions submitted between August 2020 and August 2022 were tested in parallel with the reference assay and were separated into two populations for the statistical analysis, as described below. To further evaluate sensitivity, 42 archived serum samples from cases with associated *B. canis* positive blood culture results were evaluated.

Data from serum samples submitted for *B. canis* serologic testing from January 2023 through December 2024 were retrospectively evaluated to understand the in-use assay performance.

### 2ME-RSAT and AGID II assays

Antigens for both the 2ME-RSAT and AGID II assays were produced by growing large quantities of the *B. canis* (M-) strain on *Brucella* agar under BSL3 conditions. The plated organism was harvested, washed with PBS and heat killed at 80 °C for at least 20 min. Once the product was confirmed to be non-viable, it was relocated to the laboratory for further processing. The antigen used for slide agglutination was washed with PBS, stained overnight with a Rose Bengal solution, resuspended in tris-maleate buffer, and diluted to a 6% concentration. The product was then tested in comparison to the current antigen lot and aliquoted for storage. The bacterial suspension designated for AGID II antigen testing was processed through a French Press. The lysate was centrifuged at 8,000 × g, and the supernatant was further clarified by ultracentrifugation at 63,000 × g. The resulting supernatant was tested and diluted as necessary to match the results of the current in-use cytoplasmic antigen.

All diagnostic samples were tested by both the 2ME-RSAT and AGID II tests and test interpretations were based on the evaluation of both results, along with pertinent testing/health history of the animal, if provided, following established standard operating procedures. Briefly, the slide agglutination test was performed by mixing equal amounts of patient serum and 0.2 M 2-ME on a clear slide and allowing that to stand for 30 s. The *B. canis* slide antigen was then mixed with the treated sample and the slide was rocked for 3 min before reading the agglutination reaction using an inverted microscope with 4× objective. The AGID II test was performed using in-house made AGID agar and a template to punch a seven-well pattern, including a center well for the cytoplasmic antigen and six surrounding wells for alternating positive control and diagnostic samples. The wells were labeled as indicated on the worksheet template and serum was pipetted into the corresponding wells. Positive control serum was added to the indicated outer wells and the cytoplasmic antigen into the center well. The plate was incubated overnight at 25–29 °C, and the reactions were read the following day using a fiber optic light to illuminate the precipitin lines.

### Identification of *Brucella canis* immunoreactive antigens by western blotting and proteomic analysis

*Brucella canis* antigen preparation, as described above for the 2ME-RSAT, was diluted 1:2 in Laemmli sample buffer (BioRad, Hercules, CA) with 1% 2-ME (Sigma-Aldrich Inc., St. Louis, MO), boiled for 3 min, resolved on a 12% SDS-PAGE gel, and transferred to a PVDF membrane for subsequent immunoblotting. Serum from dogs that were previously positive or negative by the *B. canis* reference assay, 2ME-RSAT and AGID II (AHDC, Cornell University, Ithaca, NY) were used to probe the immunoblot. Sera were diluted at 1:400, and serum antibody binding was detected with an HRP-conjugated rabbit anti-dog IgG(H + L) (Jackson Immunoresearch Laboratories, West Grove, PA; RRID: AB_2339344), diluted 1:20,000, resolved with ECL western blotting substrate (BioRad, Hercules, CA). A prominent band of apparent molecular weight of ~28 kDa was identified on the immunoblot (data not shown). This band was extracted from a Coomassie stained gel run in parallel and processed for mass spectrometry by the Cornell University Biotechnology Resource Center. Comparison to the protein database showed the predominant protein to be a homolog of the *B. abortus* BP26 protein.

### Cloning, expression and purification of *Brucella canis* BP26 antigen

Full-length BP26 was cloned from *B. canis* genomic DNA isolated from a *B. canis* (M-) strain maintained at the AHDC. The primers used for producing the BP26 antigen described here are listed in Table 1. Full-length BP26 (753 bp, GenBank, AQNA01000002) was cloned into a pQE-60 vector (Qiagen Inc., Germantown, MD) digested with *Nco*I and *Bam*HI, in-frame with a C-terminal 6x Histidine tag. A more soluble version of the BP26 antigen, here on described as BP26, was produced by subcloning a truncated construct into the *Nco*I/*Bam*HI digested pQE-60 vector to remove the 21 amino acid N-terminal transmembrane domain, thereby improving protein expression.

**Table 1:** Primer sequences for cloning of BP26 antigen.

Antigen	Primer direction	Primer sequence
Full-length BP26	Forward^a^	5′ cgctcATGAACACTCGTGCTAGCAATTTTCTCG
	Reverse^b^	5′ gcgggatccCTTGATTTCAAAAACGACATTGACCGATACGTT
BP26	Forward^a^	5′ cgcccatggCACAGGAGAATCAGATGACG
	Reverse^b^	5′ cgcagatctCTTGATTTCAAAAACGACATTGAC
Omp31	Forward^a^	5′ aatcATGAAGTCCGTAATTTTGGCGTCC
	Reverse^b^	5′ aaagatctGAACTTGTAGTTCAGACCGACG

^a^*Bsp*HI and *Nco*I restrictions sites for cloning underlined. ^b^*Bam*HI and *Bgl*II restriction sites for cloning underlined.

The BP26 protein was expressed in the SG13009 *E. coli* expression host (Qiagen Inc., Germantown, MD), grown in LB broth containing kanamycin and ampicillin. Protein expression was induced by the addition of 1 mM isopropyl *β*-D-1 thiogalactopyranoside (IPTG). The bacteria were suspended in urea lysis buffer (100 mM sodium phosphate, 10 mM Tris, 8 M urea, pH 8.0) and subjected to sonication. The lysate was then cleared by centrifugation, mixed 1:5 with 40 mM imidazole buffer (40 mM imidazole, 20 mM sodium phosphate, 0.5 M NaCl), and filter sterilized through a 0.22 μm Steritop™ filter (MilliporeSigma, Burlington, MA). The His-tagged protein was purified on a FPLC instrument using a HisTrapFF Ni-NTA column (both, GE Healthcare, Piscataway, NJ) and eluted with 500 mM imidazole buffer (500 mM imidazole, 20 mM sodium phosphate, 0.5 M NaCl). Eluted protein fractions were dialyzed against phosphate buffered saline, pH 7.4. Protein concentrations were determined by BCA assay (Pierce, Rockford, IL).

### Identification and production of an immunoreactive region of Omp31

Additional antigen candidates were identified by searching the protein databank for immunoreactive *B. canis* proteins. One of the candidates investigated was Omp31 (ERU01676.1) (Cassataro et al., 2004). The full-length protein was cloned as described for BP26 (Table 1); however, the recombinant protein was toxic to *E. coli* in the production platform described above for BP26, and purity of the recombinant protein was difficult to establish (data not shown). This led us to investigate potential immunoreactive peptides from Omp31, starting with evaluation of potential outer-membrane loops.

A transmembrane topology prediction of Omp31 was performed. First, the signal sequence was removed, as defined by SignalP-4.1^1^ prediction. Next, the sequence was evaluated with TMpred.^2^ Based on analysis of the mean burial propensity, a region at the C-terminal domain of the protein, representing amino acids 49–77 of the Omp31 protein, was hypothesized to be an externally exposed loop that would have increased exposure for immune recognition. A peptide derived from this region (PO1, [NH2]GKFKHPFSSFDKEDNEQ[COOH]) was synthesized (Lifetein, Hillsborough, NJ), and conjugated to maleimide-activated bovine serum albumin (BSA), through a cysteine amino acid added to the C-terminus of the peptide, following manufacturer instructions (Pierce Biotechnology, Rockford, IL).

### Coupling of antigen to fluorescent beads

In preparation for the multiplex assay, BP26 was coupled to fluorescent bead 33, BSA-conjugated PO1 was coupled to fluorescent bead 34, and for the control for the three-antigen assay, cytoplasmic antigen (CytAg) produced for the AGID II assay was coupled to fluorescent bead 35 (Luminex Corp.).^3^ The coupling was performed in accordance with manufacturer recommendations using establish laboratory protocols, as previously described (Wagner et al., 2011a). Briefly, all steps were conducted at room temperature, beads were suspended by vortex and sonication, incubations were performed in the dark, and beads were pelleted by centrifugation at 4,700 × g for 4 min. Bead stock (1.0 × 10^7^ beads) were washed with sterile ultrapure water. The beads were activated by first suspending in 100 mM sodium phosphate pH 6.2. Next, 20 μL of 10% (w/v) Sulfo-N-hydroxysulfosuccinimide (Sulfo-NHS, Pierce Biotechnology Inc., Rockford, IL), followed by 20 μL of 10% (w/v) 1-ethyl-3-(3-dimethylaminopropyl)carbodiimide hydrochloride (EDC, Pierce Biotechnology Inc., Rockford, IL) were added and incubated for 20 min. The beads were then washed twice with 50 mM 2-(N-morpholino) ethanesulfonic acid buffer pH 5.0 (MES, Sigma-Aldrich Inc., St. Louis, MO). The activated beads were mixed with the MES buffer and 200 μg of the BP26, BSA-conjugated-PO1 protein, or CytAg in a 1 mL volume and rotated for 3 h to complete the coupling. Next, the beads were incubated in PBN blocking buffer (PBN, phosphate buffered saline with 0.1% bovine serum albumin and 0.5% sodium azide) for 30 min, and finally the beads were washed three times in PBN blocking buffer with 0.02% (v/v) Tween 20. Beads were then counted and stored in the dark at 2–8°C. Each coupling batch was tested in single-plex and compared to the previous coupling batch using previously tested negative, low positive, and high positive sera to ensure comparable results across batches.

### Multiplex assay for quantification of *Brucella canis*-specific antibodies in canine serum

Beads 33 and 34 coupled with BP26 and PO1, respectively, for the two-antigen assay, or with the control CytAg coupled bead 35 for the three-antigen assay, were sonicated, mixed, and diluted in the PBN blocking buffer, to a final concentration of 10^5^ beads/mL. Canine serum samples were diluted 1:600 in PBN. Previously tested negative, low positive, and high positive canine sera were set on each assay plate as negative and positive controls. Millipore Multiscreen HTS plates (Millipore, Danvers, MA) were wetted for 10 min with phosphate buffered saline containing 0.05% Tween 20 (PBST). An ELx50 plate washer (Biotek Instruments Inc., Winooski, VT) was used for adding and aspirating PBST for this incubation and subsequent wash steps. After aspirating PBST, 50 μL of each diluted serum or control sample was added to the appropriate wells of the plate. Next, 50 μL of the bead solution was added to each well, and the plate was incubated for 30 min, with shaking, at room temperature. After washing the serum-incubated beads three times, 50 μL of biotinylated rabbit anti-dog IgG(H + L) (Jackson Immunoresearch Laboratories, West Grove, PA; RRID: AB_2339344), diluted 1:3,500 in PBN, was added to each well and incubated for 30 min as above. Following a wash step, 50 μL of streptavidin-phycoerythrin (Invitrogen, Carlsbad, CA), diluted 1:100 in PBN, was added to each well. Plates were incubated for 30 min as above and then washed. Beads were resuspended in 100 μL of PBN and incubated for 15 min, as above. The resuspended beads were then analyzed in a Luminex 200 instrument (Luminex Corp.) (see text footnote 3) using BioPlex software (Bio-Rad Laboratories Inc., Herculese, CA). The data were reported as median fluorescent intensities (MFI).

### Statistical analysis

The performance of the two-antigen, BP26 and PO1, Canine Brucella Multiplex (CBM) assay was evaluated using a Bayesian latent class model (BLCM). This model estimates the sensitivity and specificity of both the CBM assay and the reference assay by utilizing two populations expected to have different prevalence (Cheung et al., 2021). For the model to be identifiable, the number of degrees of freedom must be greater than or equal to the number of unknown parameters in the model. In this case, including two populations with different prevalence, with the assumption that the sensitivity and specificity of the assays were constant across populations, allowed for the model to be identifiable. The two populations (Table 2) were: (1) routine North American diagnostic submissions that are typically received from veterinarians because of clinical signs consistent with *B. canis* infection or for screening prior to breeding (*n* = 1,192), and (2) North American submissions requesting documentation of freedom from infection for export of the dog or the dog’s semen from the US to a foreign country (*n* = 390). Dogs in the latter population are typically free of clinical signs related to *B. canis*. Prior information about the diagnostic specificity of the reference assay and the population prevalence were modeled using unimodal beta distribution based on published data and laboratory submission history. The specificity of the 2ME-RSAT and AGID II combination test was estimated to be >99%, with a minimum plausible value of 94% (Keid et al., 2009). Based on historic diagnostic submissions to the AHDC for *B. canis* serology, 2015–2019, the prevalence of positive results in non-export North American diagnostic samples was estimated to be 10%, with a maximum plausible value of 20%, and in export sample submissions, prevalence was estimated to be 0.01%, with a maximum plausible value of 1%. These details were used to derive parameters for prior distributions using the epi.betabuster function in the epiR package (Nunes et al., 2022) in R, version 4.2.1 (R Project, 2022). A uniform prior distribution, beta(1,1), was used for the sensitivity and specificity of the assay under investigation and for the sensitivity of the reference assay. The Bayesian model was run in R using JAGS (2022) through the R2jags package (Su and Yajima, 2021), and diagnostics were visualized using the mcmcplots and coda packages (Curtis et al., 2018; Plummer et al., 2020). To investigate the sensitivity of the model to the defined priors, the model was also evaluated with minimally informative priors, beta(1,1).

**Table 2:** Distribution of assay results by population for BLCM.

	Diagnostic submissions					Export submissions			
		Reference assay					Reference assay		
		Positive	Negative				Positive	Negative	
CBM^a^	Positive	119	113	232	CBM^a^	Positive	0	18	18
	Negative	13	947	960		Negative	0	372	372
		132	1,060	1,192			0	390	390

^a^CBM results interpreted based on the cut-off values, BP26 < 2,400, PO1 < 1,000.

ROC curve analysis was performed by evaluating the BLCM model output at 15 different sets of cut-off values for PO1 and BP26 (Supplementary Table S1). Area under the ROC curve was calculated using the trapezoid rule.

For all statistical analyses, samples that were considered “inconclusive” (*n* = 43, all from the North American diagnostic submissions) on the reference assay were handled as “positive” or “negative,” depending on the specific 2ME-RSAT/AGID II reference assay results: inconclusive samples were classified as “positive” if the 2ME-RSAT results were “positive” and the AGID II results were “suspicious” (*n* = 16), else (2ME-RSAT “negative”/AGID II “suspicious,” or 2ME-RSAT “positive”/AGID II “negative”) they were considered “negative” (*n* = 27).

## Results

### Combined detection of two immunoreactive antigens aid in the serologic diagnosis of *Brucella canis* infection

As existing laboratory methods for diagnosing *B. canis* infection pose significant practical limitations, we sought to develop and explore a novel assay using two immunogenic recombinant antigens, BP26 and PO1, derived from this zoonotic pathogen. Each antigen was evaluated in a singleplex assay and in the combined multiplex assay format with negative, low positive, and high positive sera, and no significant difference in values was observed when the beads were multiplexed. Cut-off values for each antigen were selected to optimize sensitivity and specificity (Supplementary Figure S2); samples with BP26 < 2400MFI and PO1 < 1000MFI were considered negative, and samples with values above these cut-off values were considered non-negative.

**Figure 2:**
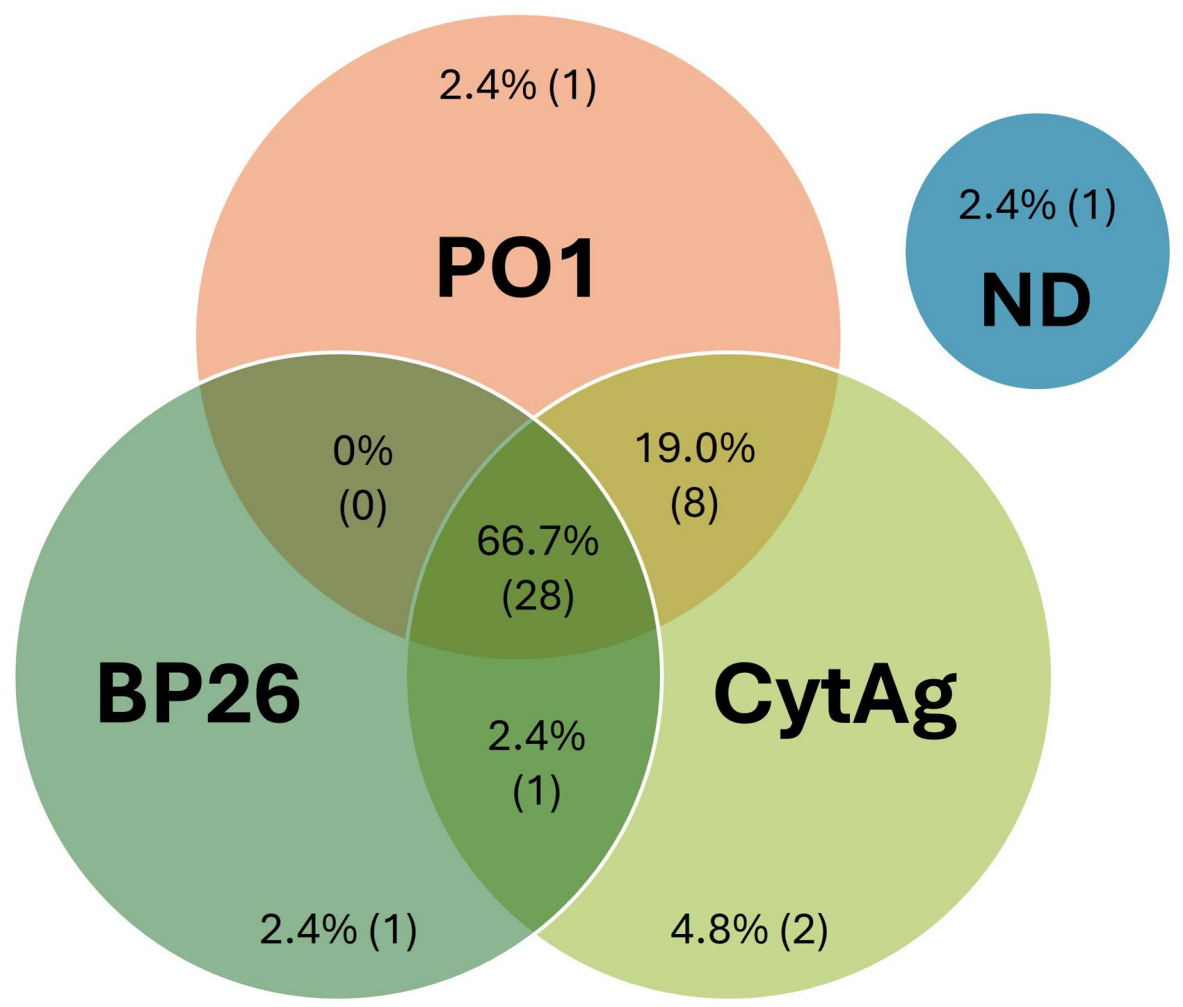
CBM assay results using serum samples from *Brucella canis* blood culture positive dogs. Serum samples from a total of 42 dogs that had a follow-up confirmatory blood culture of *B. canis.* Antibodies were considered detected if the MFI was >1,000 for PO1, >2,400 for BP26, or >1,100 for CytAg. The measured values are expressed in graphical format in Supplementary Figure S3. ND, Not Detected.

As the “gold standard” for diagnosing *B. canis* infection is bacterial culture, we evaluated archived serum samples from 42 dogs with associated *B. canis* blood culture confirmation. We found that 39 of these samples were positive on the 2ME-RSAT/AGID II reference assay, while 9 were “inconclusive,” with positive reaction on the 2ME-RSAT only, and suspect (6/9) or negative (3/9) reaction on the AGID II. Thirty-nine of the 42 serum samples were non-negative on the two-antigen CBM assay (Figure 2; Supplementary Figure S3), with 2 dogs producing only BP26 antibody values, 9 dogs producing only PO1 antibody values, and 28 dogs producing antibody values above the cut-off values for both antigens. These results pointed to individual differences of the *B. canis* antibody response in these confirmed infected dogs. Most dogs (*n* = 22) had higher PO1 than BP26 antibody values, while 17 dogs showed the opposite trend, and three dogs did not have detectable antibodies against either antigen. Together, these results demonstrated the value of including both antigens for the CBM assay to identify *B. canis* specific antibodies in dogs with confirmed infection.

### The sensitivity and specificity of the two-antigen assay was found to be reasonable

Given that the serological reference assay is not a true “gold standard” for diagnosis of *B. canis* infection, we employed a BLCM analysis to estimate the accuracy of the two-antigen CBM assay. This method allows for the estimation of the sensitivity and specificity of both the test under evaluation as well as the reference assay and estimates the seroprevalence of each population included in the model.

We used prior information about the specificity of the reference assay and the expected populations’ prevalence to help inform the model (as described in the Materials and Methods). Distributions for the prior information are presented in Supplementary Figure S1.

A ROC curve was produced by evaluating the BLCM model at 15 different sets of cut-off values (Supplementary Table S1). The area under the ROC curve (AUC) was 0.913 (95% CI, 0.883–0.917), indicating excellent to outstanding agreement (Mandrekar, 2010) between the reference assay and the two-antigen CBM assay.

The sensitivity and specificity estimations for both the two-antigen CBM assay and the reference assay at the cut-off values of BP26 < 2,400, PO1 < 1,000 is presented in Table 3. The sensitivity of the two-antigen CBM assay was estimated to be 91.6% (95% CI, 85.2–98.0%), which was substantially greater than the estimated sensitivity of the reference assay, estimated to be 66.1% (95% CI, 55.2–81.7%) (Table 3). The specificity of the two-antigen CBM assay was estimated to be 94.9% (95% CI, 92.3–96.9%), slightly lower than the estimated specificity of the reference assay 99.8% (95% CI, 99.0–100.0%). Estimated seroprevalence was determined to be 0.1% (95% CI, 0.0–0.6%) for the export sample submissions, and 16.1% (12.6–19.3%) for the routine diagnostic submissions.

**Table 3:** BLCM results for test accuracy^a^.

	Sensitivity (%)	Specificity (%)
Reference assay	66.1 (55.2–81.7)	99.8 (99.0–100.0)
CBM	91.6 (85.2–98.0)	94.9 (92.3–96.9)

^a^Diagnostic assay sensitivity and specificity are expressed as median value, with 95% credible interval.

To test the robustness of the BLCM analysis, the model with minimally informative priors (beta(1,1)) for all variables was evaluated. The minimally informed model produced comparable results, with only minor increases in the estimated population prevalence, and associated small decreases in estimated sensitivity, with increased estimates for specificity, indicating that the model results were not strongly influenced by the prior information.

These results, taken together, indicate that the novel two-antigen CBM assay composed of recombinant *B. canis* BP26 and PO1 antigens, is a robust and reliable tool for the serologic diagnosis of *B. canis* infection in dogs.

### The inclusion of an additional control bead improves assay sensitivity

The relative importance of test sensitivity and specificity is defined by a variety of factors, and in the case of diagnosis of a zoonotic pathogen in a companion animal, maximizing sensitivity to reduce false negative test results may be desired; to increase assay sensitivity, we evaluated the addition of a control bead bound to a crude antigen extract, CytAg, which is also used in the AGID II assay. Prior to inclusion in the assay, a set of 150 archived diagnostic serum samples with measurable antibody to PO1 and/or BP26 were evaluated in the CBM assay, and minimal change (%CV < 7) in the quantitative values for PO1 and BP26 was observed. A cut-off value was chosen where samples with CytAg < 1100MFI were considered negative, and samples with values above this cut-off value were considered non-negative; a sample with non-negative results on any of the three beads in the assay was considered non-negative. The inclusion of this control bead resulted in detection of two additional true positive samples, for an overall diagnostic sensitivity of the CBM assay of 97.6% (41/42) (Figure 2; Supplementary Figure S3).

While the addition of the CytAg control bead increases the diagnostic sensitivity of the assay, it also impacts the assay specificity. From January 2023 through December 2024, approximately 10,000 diagnostic samples were tested on the CBM assay, which included the two recombinant antigen beads and the control bead. In total, 1,379 samples produced non-negative results. All samples that tested non-negative were subsequently tested for confirmation on the reference assay. Of the 1,379 samples that tested non-negative, 484 were non-negative only on the CytAg. Of those, only 4.3% (21/484) were confirmed to be positive on the reference assay, and 4.3% (21/484) produced an inconclusive result on the reference assay. Overall, the addition of the CytAg control bead increases assay sensitivity but also decreases assay specificity.

## Discussion

The CBM assay is a novel fluorescent bead-based multiplex assay that simultaneously detects antibodies to two *B. canis* antigens, BP26 and PO1 peptide, to aid in the diagnosis of *B. canis* infection in dogs. These two recombinant antigens were produced in a streamlined and efficient manufacturing process that can be performed without BSL3 requirements. The CBM assay produced automated, quantitative results and provided an improved diagnostic sensitivity compared to the current 2ME-RSAT/AGID II reference assay for *B. canis* diagnosis.

The use of recombinant antigens for diagnostic serological assays typically offers greater specificity than crude antigen extracts, however, identifying antigens with optimal sensitivity can be challenging. By utilizing a bead-based multiplex platform, multiple antigens can be evaluated in parallel in the same reaction. The advantages of this platform also include a lower limit of detection, decreased background reactivity, and a broader linear range for quantification (Sipka and Wagner, 2025). The two antigens described here were specific for detecting *B. canis* antibodies in dogs, and simultaneous detection of antibodies to both antigens contributes to the enhanced sensitivity and specificity of the CBM assay. The inclusion of a crude antigen control, CytAg, does not impact the antibody values for the recombinant antigens and is useful to increase sensitivity (Figure 2; Supplementary Figure S3). Inclusion of this additional antigen does results in decreased specificity.

BP26, a 26 kDa protein isolated from *B. abortus* S19, was described more than two decades ago as a possible target for serologic diagnosis of brucellosis in a variety of *Brucella* species other than *B. canis* (Rossetti et al., 1996). It was later found that the diagnostic sensitivity of this antigen for detection of *Brucella* infection was limited and varied depending on the species infected and the strain causing infection (Xin et al., 2013). Our results reported here are the first attempt to use BP26 to diagnose *Brucella* infection in dogs. One potential limitation of the BP26 antigen is its moderate homology with the SIMPL domain-containing protein, YggE, found in species of *Ochrobactrum*. This genus of bacterium is generally not considered pathogenic, and infection with this organism could result in false-positive *B. canis* serologic responses.

The Omp31 protein has been previously investigated for its use as a component of *Brucella* vaccines (Clausse et al., 2014; Cassataro et al., 2007). The PO1 peptide was derived from a putative outer membrane loop of the Omp31 protein. Omp31 and the associated PO1 peptide are specific to *Brucella* species, including *B. canis, B. melitensis, B. suis* and *B. ovis.* However, Omp31 is missing from *B. abortus* (Cassataro et al., 2004). Our approach investigating the use of the PO1 peptide for the serologic diagnosis of *Brucella* infection is novel and highlights the benefits of pairing PO1 and BP26 for serologic *B. canis* diagnostics. Further studies are necessary to determine whether this peptide can also improve serologic diagnostic assays for *Brucella* infection in other species.

The 2ME-RSAT/AGID II reference assay is considered to have high sensitivity and specificity (Greene and Carmichael, 2012). However, as with all serological assays, it is not a true “gold standard” to confirm infection with a pathogen. More specifically, the *B. canis* reference assay can produce false-negative results early in the course of infection when antibodies are below the assay’s lower limit of detection. It may require 2 to 3 months post-infection for the 2ME-RSAT/AGID II to indicate a positive result (Wanke, 2004). The reference assay result can also be false-positive, e.g., if antibodies are maintained in serum long after disease clearance. The results of the 2ME-RSAT will remain positive for approximately 3 months after the animal is abacteremic, and the AGID II assay may remain positive for up to 3 years after a dog has cleared infection (Wanke, 2004). These challenges of antibody detection for the purpose of diagnosing infection cause statistical uncertainty in the measurement of sensitivity and specificity of serological assays. The BLCM analysis accounts for some of these challenges by estimating a sensitivity and specificity for both the reference assay and the CBM assay, as previously shown for other serological assays (Wagner et al., 2011b). The analysis revealed a lower-than-expected sensitivity of the reference assay of 66.0% (95%CI, 55.2–81.7) in comparison to the novel two-antigen CBM assay, with no overlap in the 95% credible intervals. The sensitivity of the two-antigen CBM assay was estimated to be 91.6% (95% CI, 85.2–98.0), and this estimate closely matched the results in culture positive dogs, 92.9% (39/42). As expected, the reference assay demonstrated >99% specificity; the specificity of the two-antigen CBM assay was >92.3%.

In the United States, a limited number of screening assays are available for serologic detection of *B. canis* infection. A recent study evaluated the performance characteristics of the available *B. canis* screening assays, including a lateral flow test, an IFA, and an ELISA, as compared to CBM, 2ME-RSAT, and AGIDII (LeCuyer et al., 2025). That study revealed that all evaluated screening assays had excellent sensitivity as compared to the reference assays, but the specificity of those screening assays, in particular the ELISA and IFA, support the need for follow-up evaluation to confirm non-negative results.

It is typically recommended that all *B. canis-*infected dogs are spayed/neutered or euthanized. When treatment is elected over euthanasia, continued monitoring of antibody values during and after treatment may be appropriate. Antimicrobial therapy will reduce bacteremia along with a corresponding decrease in antibodies over time, however bacteremia can rebound after treatment is discontinued (Greene and Carmichael, 2012). Bacterial recrudescence can occur in dogs without clinical signs of disease and poses a potential public health threat. Continued serological monitoring with a sensitive and quantitative test, such as the CBM assay, may be beneficial for providing evidence for recrudescence. A recent study evaluating the use of the CBM assay for monitoring response to treatment in *B. canis* infected dogs revealed an association between decrease in PO1 antibody values and resolution of clinical signs (Guarino et al., 2023).

The primary limitation of this study is the use of diagnostic submissions from dogs with unknown clinical status and history for the BLCM analysis. Further, inclusion of a very low prevalence population in the BLCM analysis introduces a larger uncertainty in sensitivity measurement, however, the evaluation of a set of 42 serum samples from animals confirmed to be infected with *B. canis* corroborates the sensitivity results obtained for the CBM assay. Additional limitations of this study are related to the assumptions inherent to the BLCM analysis, these include: (i) sensitivity and specificity of the assays were constant across populations, and (ii) the CBM and reference assay results were independent, conditional on true disease state. Test sensitivity and specificity values can be impacted by characteristics of the population, including factors such as infection pressure (Leeflang et al., 2013) or presence of cross-reacting agents (Greiner and Gardner, 2000). For these reasons, samples submitted from foreign countries, where infection pressures and the presence of cross-reacting agents may vary, were not included in the BLCM analysis. Regarding conditional dependance, two tests that measure antibody response would typically be considered conditionally dependent. However, the assays used here measured antibody to different antigens. The reference assay measures antibodies against cytoplasmic antigens (AGID II) and/or cell surface antigens (2ME-RSAT), while the CBM assay detects antibody against a periplasmic protein (BP26) and a portion of an outer membrane protein (PO1). Even so, it may be appropriate to consider the latent class in this model as antibody production, rather than disease status, as all assays evaluated in this study require antibody production to produce a positive result. Further, if the bacteria become sequestered in a region of the body (e.g., eye, central nervous system, testis), and the peripheral immune system is no longer stimulated, antibody production can cease, leading to negative serology in an infected animal. Regardless of these limitations, the agreement between the reference assay and the CBM assay is excellent, as evidenced by AUC > 0.8.

In conclusion, the CBM assay is a robust, reliable, and quantitative assay to detect *B. canis* antibodies in canine serum and to aid in the diagnosis of *B. canis* infection. While the two-antigen CBM assay is both sensitive and specific, inclusion of the CytAg control bead further enhanced assay sensitivity. Confirmation of infection is still warranted in many cases, through reference assay testing and/or attempted culture of the organism, as a decision of euthanasia should not be made based on the result of any one serologic assay. Identification of additional *Brucella-*specific recombinant antigens that would replace the CytAg in the multiplex assay could further enhance the accuracy of the CBM assay. Further studies are ongoing to evaluate the use of the CBM assay for infection confirmation, monitoring response to treatment, detection of infection with other *Brucella* spp., and disease surveillance.

## Data Availability

The original contributions presented in the study are included in the article/Supplementary material, further inquiries can be directed to the corresponding author.

## Footnotes

^1^ http://www.cbs.dtu.dk/services/SignalP-4.1/

^2^ https://embnet.vital-it.ch/software/TMPRED_form.html

^3^ http://www.luminexcorp.com
