# Supplementary material for: A fluorescent bead-based multiplex assay for the detection of Brucella sp. specific antibodies in canine serum
Source: Front Microbiol. 2025 Oct 8;16:1655877. doi: 10.3389/fmicb.2025.1655877 (PMC12540404; doi:10.3389/fmicb.2025.1655877)
Supplement: Supplementary file 1 [file Data_Sheet_1.docx]

Table S1. Cut-off values used to produce ROC curve

| PO1 MFI | BP26 MFI |
| --- | --- |
| 100 | 240 |
| 150 | 350 |
| 200 | 500 |
| 350 | 750 |
| 500 | 1000 |
| 650 | 1500 |
| 850 | 2000 |
| **1000** | **2400** |
| 1500 | 3000 |
| 2000 | 3600 |
| 3500 | 4200 |
| 5500 | 5500 |
| 10000 | 10000 |
| 15000 | 15000 |
| 20000 | 20000 |


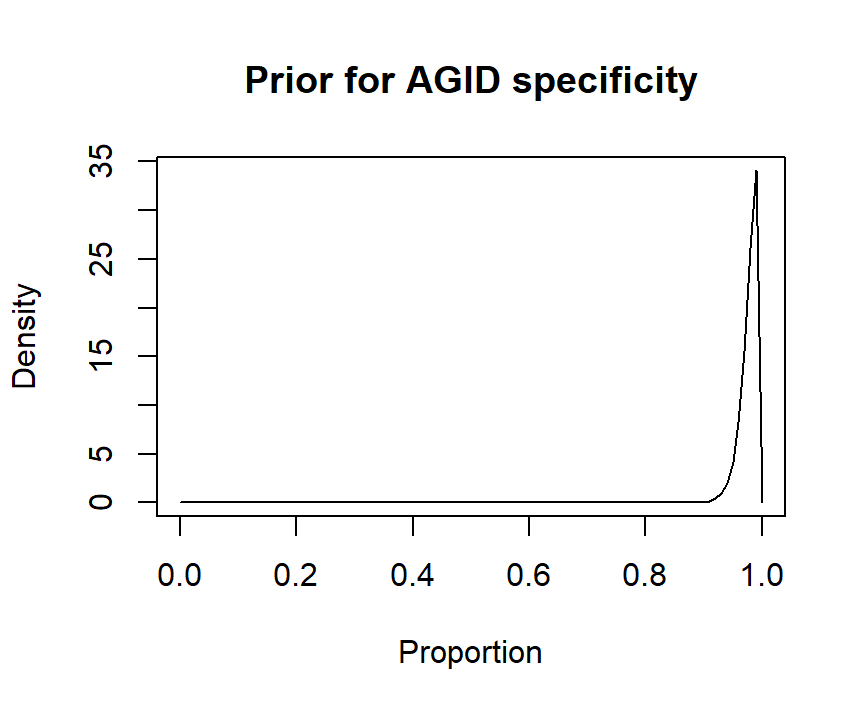

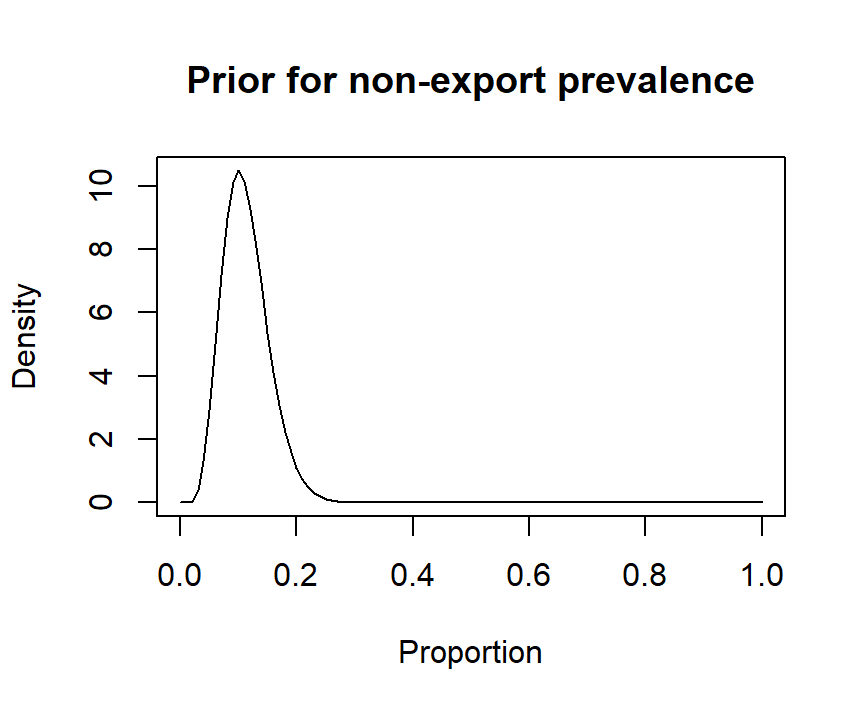


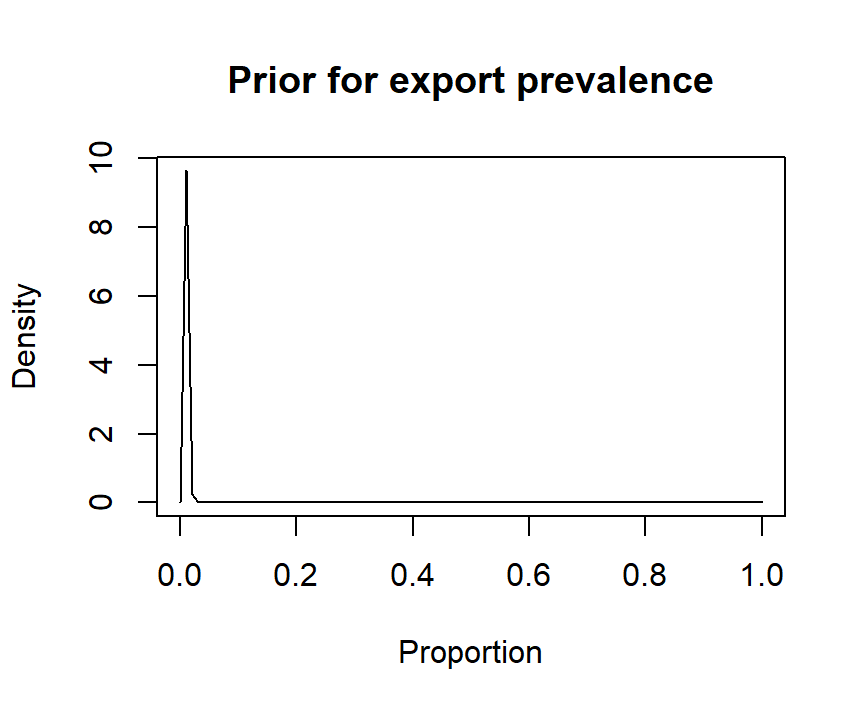


**Figure S1. Prior distributions for population prevalence and reference assay specificity.**

**Figure S2. ROC curve.** Receiver operating characteristic curves (ROC) were produced by evaluating sensitivity and specificity of the Canine Brucella Multiplex assay at 15 different sets if cut-off values using the BLCM model described in the text. The upper and lower lines represent the 95% CI, and the highlighted points represent the chosen cut-off values of 1000 MFI and 2400 MFI for PO1 and BP26, respectively.

**Figure S3. Quantitative results from culture confirmed cases.** Serum samples from a total of 42 dogs that had a follow-up confirmatory blood culture of *B. canis.* Serum from dogs #18, 19, and 21 were negative on AGID II. Sera from dog #3 was negative on 2ME-RSAT. Bars represent the standard deviation of three independent replicates.
