# Supplementary material for: A fluorescent bead-based multiplex assay for the detection of Brucella sp. specific antibodies in canine serum
Source: Front Microbiol. 2025 Oct 8;16:1655877. doi: 10.3389/fmicb.2025.1655877 (PMC12540404; doi:10.3389/fmicb.2025.1655877)
Supplement: Supplementary file 2 [file Data_Sheet_2.docx]

**Additional Supplementary Material**

The model used for the BLCM analysis is provided below.

model{

#=== LIKELIHOOD ===#

#=== POPULATION 1 ===#

Pop1[1:4] ~ dmulti(p1[1:4], 1192)

p1[1] <- Prev1*Se_BacT*Se_Multi + (1-Prev1)*(1-Sp_BacT)*(1-Sp_Multi)

p1[2] <- Prev1*Se_BacT*(1-Se_Multi) + (1-Prev1)*(1-Sp_BacT)*Sp_Multi

p1[3] <- Prev1*(1-Se_BacT)*Se_Multi + (1-Prev1)*Sp_BacT*(1-Sp_Multi)

p1[4] <- Prev1*(1-Se_BacT)*(1-Se_Multi) + (1-Prev1)*Sp_BacT*Sp_Multi

#=== POPULATION 2 ===#

Pop2[1:4] ~ dmulti(p2[1:4], 390)

p2[1] <- Prev2*Se_BacT*Se_Multi + (1-Prev2)*(1-Sp_BacT)*(1-Sp_Multi)

p2[2] <- Prev2*Se_BacT*(1-Se_Multi) + (1-Prev2)*(1-Sp_BacT)*Sp_Multi

p2[3] <- Prev2*(1-Se_BacT)*Se_Multi + (1-Prev2)*Sp_BacT*(1-Sp_Multi)

p2[4] <- Prev2*(1-Se_BacT)*(1-Se_Multi) + (1-Prev2)*Sp_BacT*Sp_Multi

#=== PRIOR ===#

Prev1 ~ dbeta(7.185, 56.665) ## Prior for Prevalence in population 1

Prev2 ~ dbeta(1.037, 370.962999999999) ## Prior for Prevalence in population 2

Se_BacT ~ dbeta(1, 1) ## Prior for Se of Test A

Sp_BacT ~ dbeta(85.471, 1.85324242424242) ## Prior for Sp of Test A

Se_Multi ~ dbeta(1, 1) ## Prior for Se of Test B

Sp_Multi ~ dbeta(1, 1) ## Prior for Sp of Test B

}
